# Decreased Conflict Control in Overweight Chinese Females: Behavioral and Event-Related Potentials Evidence

**DOI:** 10.3390/nu11071450

**Published:** 2019-06-27

**Authors:** Yong Liu, Huan Quan, Shiqing Song, Xuemeng Zhang, Chao Yang, Hong Chen

**Affiliations:** 1 Key Laboratory of Cognition and Personality (Ministry of Education), Southwest University, Chongqing 400715, China; 2 School of Psychology, Southwest University, Chongqing 400715, China; 3 Department of Psychology, Wichita State University, Wichita, KS 67260, USA

**Keywords:** overweight, conflict control, event-related potentials, food-related stimuli

## Abstract

Overweight or obesity is related to a decrease in cognitive control, especially conflict control. However, research on conflict control in overweight/obese individuals are still controversial. This study was conducted to explore general and food-related conflict control in overweight Chinese females (OWs) with a color–word Stroop task and a food-related conflict task. Event-related potentials (ERPs) were recorded during the food-related conflict task. Behavioral results showed that, OWs had a longer reaction time (RT) than normal-weight Chinese females (NWs), in both tasks. ERP results in the food-related conflict task showed that there was a reduction of N2 and N450 response strength in OWs, and the P3 and late positive component (LPC) response strength was enhanced. Results indicated that OWs might be less efficient in monitoring and resolving conflict, and OWs tended to have a higher motivational or emotional involvement in processing food-related stimuli, which was likely to contribute to their difficulty in losing weight.

## 1. Introduction

Obesity has become one of the most prevalent problems around the world and has posed a serious threat to public health [1,2]. In China, the rate of overweight and obese individuals has been increasing rapidly. China’s obese population (89.6 million) has surpassed that of the United States since 2014, ranking first in the word [3]. Obesity not only increases the risk of physical illnesses, such as type II diabetes, hypertension, hyperlipidemia, coronary heart disease, cancer, gallbladder disease, and stroke [4], but also the risk of mental health problems, such as age-related cognitive decline [5], and even neurodegenerative diseases, such as dementia [6]. Obesity is also likely to impair the normal functions of organs, such as the heart, the skeletal muscle, and the liver [7].

It is widely acknowledged that overeating plays an important role in leading to an imbalance between motivation and control/inhibition systems [8,9]. Therefore, overweight and obese individuals might be characterized by an enhanced motivation system and a decreased control system. Cognitive control, such as conflict control, has become a hot topic of research in overweight and obese individuals. Cognitive conflicts appear with exposure to different simultaneous characterizations that interfere with each other. Conflict control refers to monitoring and controlling conflicts that occur during the processing of information [10]. Color–word Stroop task uses the incongruence of color and word to produce conflict (e.g., “red” written in blue), which is usually used to measure the general conflict control. Research using color–word Stroop task found that overweight and obese individuals had poorer general conflict control performance than normal-weight individuals [11,12,13,14]. In addition, similar findings were found in other studies that applied Go/No-Go, Flanker, and Stop signal tasks to examine the general conflict control in overweight and obese individuals [15,16,17,18]. Go/No-Go task consists of Go stimuli and No-Go stimuli. Participants are asked to press a button as soon as possible during Go stimuli but to inhibit responses during No-Go stimuli. The procedure of Stop signal task is similar to Go/No-Go task, which consists of Go stimuli and Stop stimuli. Participants are asked to press a button as soon as possible during Go stimuli but to inhibit responses during Stop stimuli. The Flanker task is similar to the Stroop task, which consists of congruent and incongruent stimuli. A previous study using food-related Go/No-Go task found that overweight adolescents had a poorer food-related conflict control performance than normal-weight controls [19]. Moreover, a meta-analysis study found that overweight and obese individuals showed a decrease in conflict control, which did not differ in age and gender [20]. However, there are still some studies which failed to reveal an association between overweight/obesity and poorer conflict control. For instance, many studies using the Stroop task did not find the group difference between overweight/obesity and normal-weight individuals [21,22,23,24]. Therefore, it is necessary to provide further evidence for the relationship between overweight/obesity and conflict control.

Moreover, the neural mechanisms underlying the relationship between conflict control and overweight remains poorly understood. Chen et al. (2018) [17] explored the neural markers of general conflict control in both obese and normal-weight adolescents, and found that the obese adolescents had greater No-Go N2 amplitude compared to the Go N2 amplitude. Kamijo et al. (2012) [18] found that obese participants had a smaller error-related negativity (ERN) amplitude than normal weight participants, during a general Flanker task, which indicated that obese participants were characterized by a decreased ability of cognitive control. Nijs et al. (2010) [25] used food-related words Stroop task to explore the food-related conflict control in individuals with obesity, where participants were asked to ignore the content of the words and respond as quickly as possible to the font color of the words. They found that there was an enhanced P2 in obese participants, which might indicate that obese individuals displayed an enhanced, automatic, and preconscious attentional processing of food-related stimuli. Carbine et al. (2018) [26] examined the neural correlates of food-related inhibition among normal-weight, overweight, and obese adults in Go/No-Go tasks. The findings showed that No-Go trials elicited greater N2 amplitudes than Go trials in all participants. N2 is a negative potential occurring at about 200–350 ms after the onset of stimuli [26,27], reflecting sensory processing, conflict monitoring, and response inhibition, based on stimuli and tasks [27]. N450, which could be elicited by conflict information, is also a commonly used indicator in the study of conflict control. N450 is a negative component that appears 400–600 ms after stimuli presentation, reflecting conflict detection [28], conflict processing, and conflict adaptation [29]. N450 is likely to reflect activity of neural generators localized in the dorsal anterior cingulate cortex, which is involved in detection of conflict in performance or environment, as well as subsequent behavioral adjustment [30].

P3 is a positive potential occurring about 300–600 ms after the onset of stimuli [31]. P3 also reflects different cognitive processes based on tasks, such as working memory and attention allocation [32]. Overweight/obese children showed decreased P3 amplitudes during general Go/No-Go tasks, compared to normal-weight children, which suggested that overweight children had greater difficulties in processing cognitive demands [14]. Recently, some studies found in a Go/No-Go task, No-Go trials elicited a greater P3 than Go trials in both overweight/obese and normal-weight individuals [17,26]. Therefore, the group difference of P3 between overweight/obese and normal-weight individuals is still controversial and further investigation is needed. Meanwhile, a late positive component (LPC) is sensitive to the engagement of controlled cognitive resources, and it reflects higher-order cognitive processes, such as finer evaluation of stimulus meaning and response decisional processing [33]. Increased LPC reflects more resources of attention allocated to the stimuli processing, which involves motivation or emotion [34]. To our knowledge, LPC is less often applied to conflict control study in the overweight/obese. The present study aimed to examine both early and late processing of stimuli in overweight and normal-weight adults. Therefore, we explored not only the early event-related potentials (ERPs), but also the late ERPs.

The aim of the current study was to extend previous work on general and food-related conflict control by comparing both behavioral and neurocognitive correlates in the overweight and normal-weight Chinese females. We used a color–word Stroop and a food-related conflict task to explore general conflict control and food-related conflict control, respectively, and ERPs were recorded during the food-related conflict task. Behavioral and electrophysiological methods were used to assess aspects of conflict control in overweight Chinese females (OWs) and normal-weight Chinese females (NWs). Based on previous studies, we hypothesized that OWs might have a lower level of ability to detect conflicts, which is manifested by a longer reaction time (RT), lower accuracy (ACC), and decreased N2, N450; OWs might also allocate more cognitive resource to food-related stimuli, which is reflected by an enhanced P3, LPC.

## 2. Methods

### 2.1. Participants

Chinese female participants (*N* = 43) were recruited through campus advertisements at Southwest University in China. Only female adults were recruited because men and women differ in how and why they gain and lose their weight [35]. Participants with a BMI equal to or greater than 25 kg/m^2^ (M = 28.84, SD = 3.86) were included in the overweight group (*N* = 22), and those with a BMI between 18 and 22 kg/m^2^ (M = 20.81, SD = 1.49) were assigned to the normal weight group (*N* = 21). None of them reported having physical or psychiatric problems that might interfere with the current study. Before starting the experiment, all participants read the instructions and could ask questions about the experiment before signing the consent to participate. For standardization purposes, all participants were asked to abstain from consuming any food except for water, for at least four hours before the experiment [36,37]. The current study was approved by the Southwest University Ethics Committee.

### 2.2. Self-Report Measures

#### 2.2.1. Hunger, Desire to Eat, and Body Satisfaction

Participants rated their hunger, desire to eat, and body satisfaction on a 100 mm visual-analog scale (VAS), ranging from “not at all” to “very much.”

#### 2.2.2. Negative Physical Self Scale—Fatness Concern (NPS–F)

The NPS–F [38] is an 11-item questionnaire that assesses physical self-perception regarding fatness. Participants were asked to rate their responses to certain statements (e.g., “My weight has always been a pain in my heart”) on a 5-point Likert scale from 0 (never) to 4 (always). The original scale has a Cronbach’s alpha coefficient of 0.88; in the current study, NPS–F had a Cronbach’s alpha coefficient of 0.93.

#### 2.2.3. Dutch Eating Behavior Questionnaire—Restraint Scale (DEBQ–R)

The DEBQ–R [39] is a 10-item scale that assesses dietary behaviors related to the loss and maintenance of weight (e.g., “Do you take into account your weight with what you eat?”). For each item, respondents rated their degree of restrained-eating on a 5-point Likert scale from 1 (never) to 5 (always). Higher DEBQ–R scores indicates more restrained-eating. The scale had a good internal consistency in general, with alphas coefficient ranging from 0.93 to 0.95. Its two-week test-retest reliability was 0.82. In the current study, the DEBQ–R had an alpha coefficient of 0.89.

### 2.3. Experimental Tasks

#### 2.3.1. Color–Word Stroop Task

**Figure 1:**
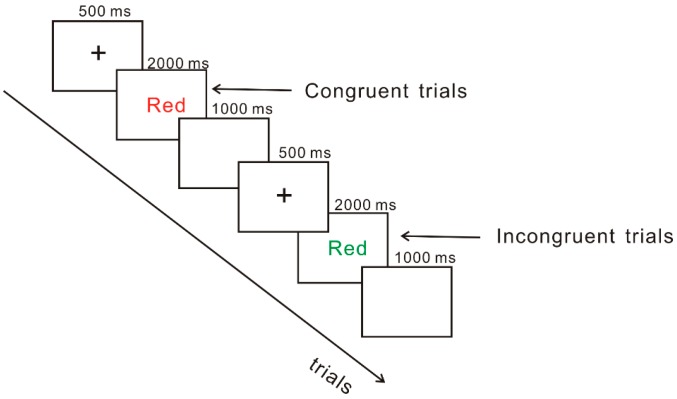
Depiction of a congruent and an incongruent trial in the color–word Stroop task.

In the color–word Stroop task, stimulus was presented on a computer screen. Participants responded with the computer keyboard. The targets were the Chinese words ‘red’, ‘green’, ‘yellow’, and ‘blue’ displayed in the red, green, yellow, and blue color. Congruent (e.g., the Chinese word ‘red’ displayed in red) and incongruent (e.g., the Chinese word ‘red’ displayed in green, yellow, or blue) trials were produced by a combination of color words and color. Four keys on the computer keyboard were covered with different color patches; participants were told to press the key that corresponded to the color of the target as soon as possible. The task consisted of two runs. One run asked participants to response to the word, and the other one asked participants to response to color. Run order was counterbalanced across participants. Each run consisted of a practice block of 20 trials, followed by an experimental block of 60 trials. Each trial began with a fixation point presented for 500 ms in the center of the screen, after which the stimulus was presented on the screen until participants gave response. If participants did not respond, stimuli would automatically disappear after 2,000 ms. In order to eliminate the interaction between stimuli, as soon as possible, an inter-stimuli interval of 1,000 ms was presented (Figure 1).

#### 2.3.2. Food-Related Conflict Task

**Figure 2:**
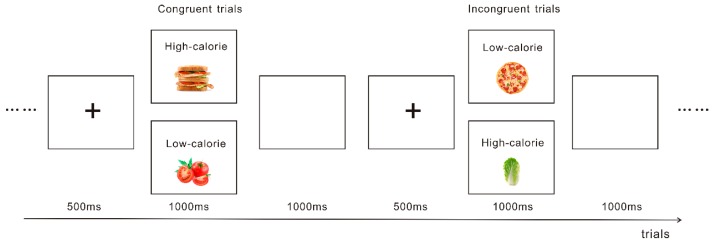
Depicts examples of stimuli from the food-related conflict task.

The food-related conflict control task used in the current study was newly designed. In the task, the stimuli consisted of food pictures (including high- and low-calorie food) and the words ‘high-calorie’ or ‘low-calorie’. Congruent and incongruent trials were produced by a combination of food pictures and calorie information. For example, we combined high-calorie food pictures with the words ‘high-calorie’ to form the congruent trials. Food pictures used in the task were taken from the ones previously used in our studies [40,41]. Each trial began with a fixation point presented for 500 ms in the center of the screen, after which the stimuli were presented on the monitor until the participants gave a response. If participants did not respond, the stimuli would automatically disappear after 1,000 ms, followed by an inter-stimuli interval of 1,000 ms (Figure 2). The task consisted of a practice block of 20 trials, followed by an experimental block of 200 trials. During the task, participants were asked to press ‘F’ (or ‘J’) for the congruent trials and ‘J’ (or ‘F’) for the incongruent trials, and responses were counterbalanced across participants.

During the food-related conflict task, event-related potentials (ERPs) were recorded from 64 scalp sites, using tin electrodes mounted on an elastic cap (Brain Products GmbH, Gilching, Germany), with the reference electrodes placed on FCz (frontocentral aspect) and a ground electrode on AFz (medial frontal aspect). The vertical electrooculogram (IO) was recorded with an electrode placed infra-orbitally at the right eye. All inter-electrode impedance was maintained below 5 KΩ.

### 2.4. Behavioral Analyses

Independent sample *t*-tests were conducted to identify between-group differences in age, BMI, hunger, desire to eat, body satisfaction, NPS–F, and DEBQ–R.

For the color–word Stroop task, 2 (group—overweight and normal weight) × 2 (condition—congruent and incongruent trials) × 2 (run—response to word and color) three-way ANOVAs was conducted on reaction time (RT) for correct responses and ACC, with group as a between-group factor, and condition and run as the within-group factors.

For the food-related conflict task, 2 (group—overweight and normal weight) × 2 (condition—congruent and incongruent trials) two-way ANOVAs was conducted on RT for correct responses and ACC, with group as a between-group factor and condition as a within-group factor.

### 2.5. ERPs Recording and Analyses

ERPs data processing was performed with EEGLAB [42], an open source toolbox running on the Matlab software. Individual and grand ERP averages were created for the food-related conflict task. We first downsampled the data from 1,000 Hz to 256 Hz and performed high-pass filtering at 0.1 Hz and low-pass filtering at 45 Hz. We selected the mean values of the left and right mastoids as the re-reference. Data were epoched from 200 ms prior to stimuli onset to 800 ms after presentation, and were baseline corrected to the pre-stimuli interval. Trials with large fluctuations in amplitudes were removed before independent component analysis (ICA). Then, the components including EOG artifacts (ocular movements and eye blinks) and head movement were removed from the results of ICA. Based on the topographical distribution of the grand-averaged ERP activities, the ERPs and their time epochs were as follows—N2 (300–360 ms), P3 (360–400 ms), N450 (400–450 ms), and LPC (500–800 ms). The following electrode sites were selected, frontal (F3, Fz, F4), frontal–central (FC3, FCz, FC4), central (C3, Cz, C4), central–parietal (CP3, CPz, CP4), and parietal (P3, Pz, P4). Four 2 (group—NWs, OWs) × 2 (condition—congruent and incongruent trials) × 5 (electrode site—frontal, frontocentral, central, central-parietal, and parietal) repeated measures ANOVAs were conducted on the mean amplitudes of N2, P3, N450, and LPC. All analyses were conducted by SPSS 22.0. *p*-value was computed for deviation in all analyses, based on the Greenhouse–Geisser method. Post-hoc *t*-tests were conducted with Bonferroni correction for multiple pairwise comparisons.

## 3. Results

### 3.1. Self-Report Results

Participants’ demographic information and self-report results are shown in Table 1. OWs had a significantly higher BMI than NWs, *p* < 0.01. The score of NPS–F in OWs was significantly higher than that in NWs, *t* = −2.27, *p* = 0.03, Cohen’s *d* = 0.69. The score of body satisfaction in OWs was significantly smaller than that in NWs, *t* = 4.46, *p* < 0.01, Cohen’s *d* = 1.36. There was no significant difference between groups in hunger, desire to eat, and DEBQ–R, all *p* > 0.05.

### 3.2. Behavioral Results

#### 3.2.1. Color–Word Stroop Task

**Figure 3:**
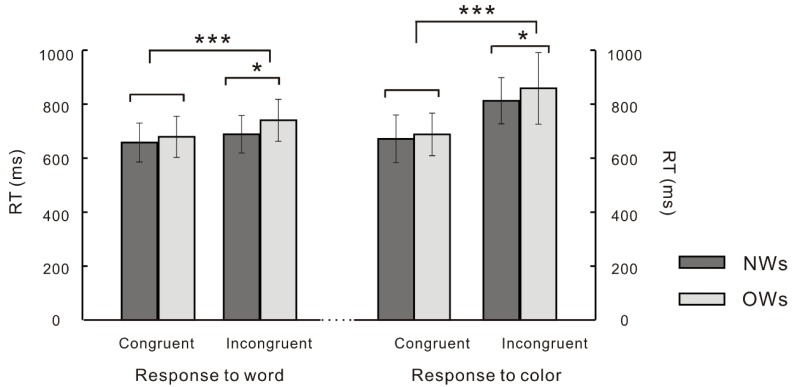
Reaction Time (RT) difference between overweight Chinese females (OWs) and normal-weight Chinese females (NWs) in the color–word Stroop task (* *p* < 0.05; *** *p* < 0.001).

Repeated-measures ANOVAs on RT (Figure 3) found a main effect of condition, F (1, 41) = 280.19, *p* < 0.001, *η^2^* = 0.87, with the post-hoc test indicating that RT of incongruent trials was longer than that of congruent trials; a main effect of run, F (1, 41) = 50.03, *p* < 0.001, *η^2^* = 0.55, with post-hoc test suggesting that RT of the run where the correct response was to the word was smaller than the run where the correct response was to color; there was also a significant interaction between group and condition, F (1, 41) = 6.168, *p* = 0.017, *η^2^* = 0.131, and a simple effect analysis showed that the RT of OWs was significantly longer than that of NWs in incongruent trials, *p* = 0.05.

**Figure 4:**
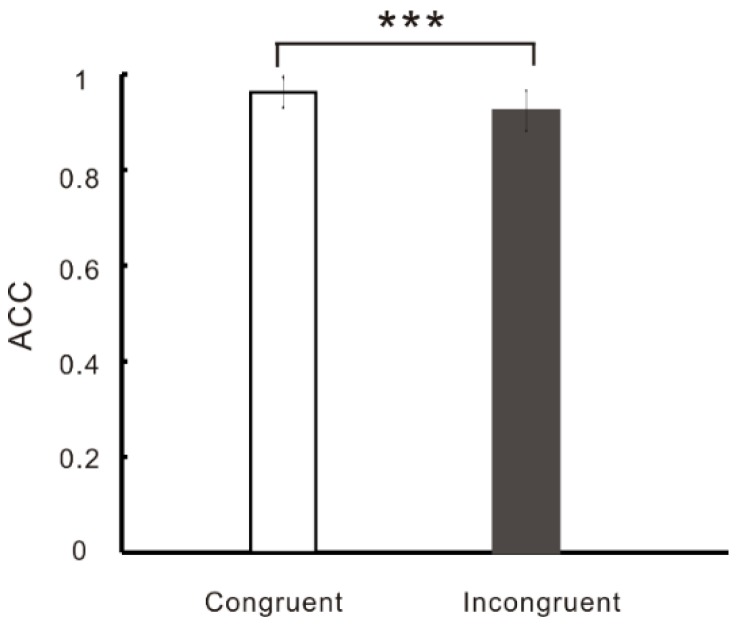
Accuracy (ACC) difference between congruent and incongruent trials in the color–word Stroop task (*** *p* < 0.001).

Repeated-measures ANOVAs on ACC (Figure 4) found a main effect of condition, F (1, 41) = 86.74, *p* < 0.001, *η^2^* = 0.68, and post-hoc test found that ACC of incongruent trials was smaller than that of congruent trials. However, we did not find the group difference between OWs and NWs.

#### 3.2.2. Food-Related Conflict Task

**Figure 5:**
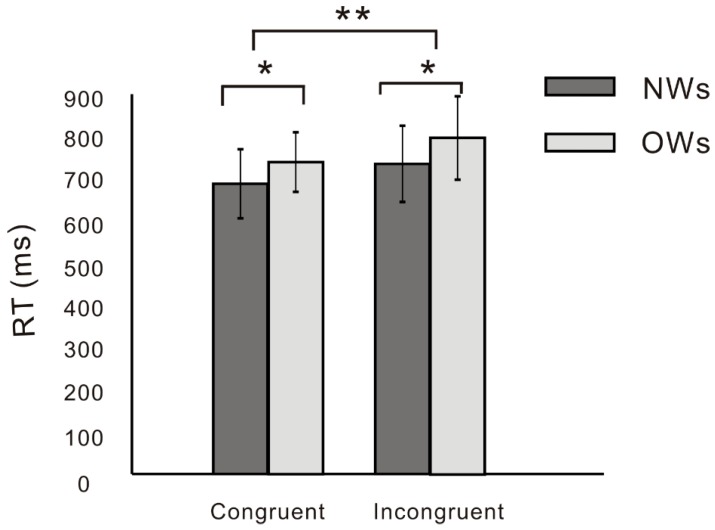
RT difference between OWs and NWs in food-related conflict task (* *p* < 0.05; ** *p* < 0.01).

Repeated-measures ANOVAs on RT (Figure 5) found a main effect of condition, F (1, 41) = 5.24, *p* = 0.03, *η^2^* = 0.113, and a post-hoc test on condition found that the RT of incongruent trials was longer than that of congruent trials. Post-hoc test on group found that RT in OWs was significantly longer than that in NWs, *p* = 0.03.

### 3.3. ERPs Results

**Figure 6:**
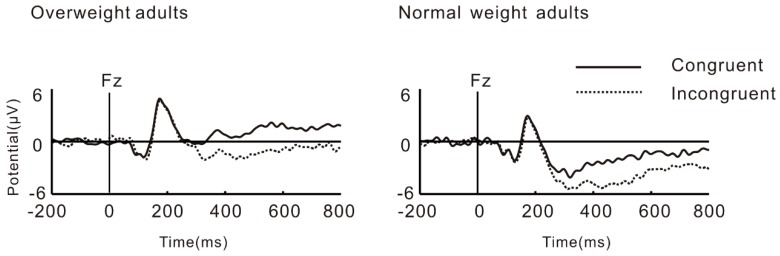
Stimuli-locked, grand average waveforms of N2, P3, N450, and late positive component (LPC) at site Fz.

Grand average ERPs for N2, P3, N450, and LPC at Fz are shown in Figure 6.

#### 3.3.1. N2

Results of repeated-measures ANOVAs on N2 showed a marginally significant main effect of condition, F (1, 41) = 3.83, *p* = 0.057, *η^2^* = 0.06, and the post-hoc test on condition found that the N2 amplitude of incongruent trials was significantly greater than that of the congruent trials; Post-hoc test on group found that N2 amplitude in OWs was significantly lower than that in NWs, *p* = 0.01.

#### 3.3.2. P3

Results of repeated-measures ANOVAs on P3 showed a significant interaction effect between group and electrode site, F (1, 41) = 4.09, *p* = 0.003, *η^2^* = 0.09, and the simple effect analysis showed that the OWs elicited greater P3 in the frontal, frontocentral, and central regions than the NWs, all *p* < 0.05. Post-hoc test on group found that P3 amplitude of OWs was significantly greater than that of NWs, *p* = 0.02.

#### 3.3.3. N450

Results on N450 found a main effect of condition, F (1, 41) = 10.04, *p* = 0.003, *η^2^* = 0.20, and the post-hoc test found that the incongruent trials elicited a greater N450 than the congruent trials; an interaction effect of the group and electrode site was significant, F (1, 41) = 4.51, *p* = 0.028, *η^2^* = 0.10, and a simple effect analysis found that the OWs elicited lower N450 than the NWs in the frontal and frontocentral regions.

#### 3.3.4. LPC

Results on LPC found an interaction effect of group and electrode site, F (1, 41) = 4.92, *p* = 0.02, *η^2^* = 0.11. A simple effect analysis found that LPC amplitude of OWs was significantly greater than that of NWs in the frontal, frontocentral, and central regions.

## 4. Discussion

In our novel examination of general and food-related conflict control in OWs and NWs, the findings partially supported our hypotheses. There was no difference in hunger, desire to eat, or DEBQ–R between OWs and NWs, which indicated that the difference in weight between the two groups was not influenced by these factors. Both in the general and food-related tasks, RT difference between the OWs and the NWs was found in the study. More importantly, evidence from ERPs also supported the behavioral results, which showed that N2 and N450 of OWs were significantly lower than those of NWs, but P3 and LPC of OWs were significantly greater than those of NWs.

Previous studies have shown that overweight/obese individuals have a longer RT to food-related words, which suggests that there is attentional bias towards food-related words in overweight/obese individuals [25,43]. In addition, researchers used a verbal and a spatial version of the Stroop task and found that there was group difference in the verbal Stroop task between the overweight and normal-weight individuals [44], which might suggest that the OWs’ ability to monitor verbal conflict is more easily damaged. In our study, RT in OWs was significantly longer than that in NWs, which suggested that conflict control was decreased in the OWs, compared to the NWs.

There was a reduction of N2 neural response strength among OWs. Folstein and Van (2008) [27] believed that N2 can be divided into three subcomponents—a frontocentral (anterior) N2 related to the detection of novelty or mismatch with a perceptual template when the eliciting stimuli are presented, a second frontocentral N2 related to cognitive control (including response inhibition, response conflict, and error monitoring), and a posterior N2 related to visual attention. N2 has been thought to reflect the detection and monitoring of response conflict between erroneous and correct responses [27,45]. The reduced N2 effect has also been attributed to a poor recruitment of neural resources [46]. Therefore, a speculative interpretation of N2 might suggest that OWs are less capable of recruiting attentional and neural resources to detect conflict over general and food-related stimuli or have less efficient conflict monitoring than NWs. In the conflict control studies, the function of N450 is similar to N2. N450 reflects conflict processing and conflict adaptation [28], which also represents the ability to inhibit and resolve conflict information [27]. In our task, when food stimuli arose, participants were asked to monitor and adapt food stimuli as soon as possible, and then respond differently to food stimuli. The reduction of N450 in OWs suggested that OWs were less capable of processing and resolving conflict stimuli.

P3 increases with task difficulty and effort [47], and it is closely related to the intensity of processing [48]. In addition, salient and self-relevant environmental cues also led to P3 enhancements [49]. Moreover, compared with neutral stimuli, food stimuli would elicit greater P3 [50], and high-calorie food would elicit greater P3 than low-calorie food [26], indicating that participants needed to allocate more resources to food stimuli, especially high-calorie food. Our results showed an enhanced P3 in OWs compared to NWs, which was inconsistent with a previous study [14], which found that overweight/obese children showed decreased P3 amplitudes. We believed that the difference might have resulted from experimental stimuli. OWs should allocate more cognitive resources to food-related stimuli. LPC is also related to the allocation of cognitive resources, but it reflects higher-order cognitive processes, such as evaluation of stimuli meaning and response decision processing [33]. Increased LPC reflects the allocation of more attentional resources in the processing of stimuli involving motivation or emotion [34]. Therefore, enhanced P3 and LPC in OWs could be interpreted in terms of the relative salience of the food stimuli that demand more cognitive resources, and OWs’ processing of food stimuli involves motivation or emotion.

In the current study, time windows of N2, P3, N450, and LPC were 300–800 ms. When food-related conflict stimuli appeared, N2 was responsible for monitoring the conflict stimuli, P3 was responsible for allocating cognitive resources for processing conflict stimuli, and N450 was responsible for adapting and resolving the conflict stimuli. Finally, LPC reflected late higher-order processing of stimuli involving motivation or emotion. To our knowledge, this was the first study investigating the general and food-related conflict control from the perspective of the course of time. It explored the dynamics of conflict control in OWs and NWs, over time. Both behavioral and ERP evidence indicated a decreased conflict control in OWs.

Conflict control is the first phase of cognitive control, and it is necessary for successful self-control [51,52]. The goal conflict model of eating suggests that the food intake of dieters is characterized by a conflict between two chronically accessible incentives or goals—eating enjoyment and weight control. Their difficulty in weight control is due to their behavioral sensitivity to eating enjoyment and its incompatibility with the eating control goal [53]. In the face of food temptation, successful dieters realize that food enjoyment is in conflict with the long-term weight control, and thus, they would show a weight-control goal. Therefore, the explorations of conflict control in OWs could provide a theoretical basis for intervention strategies targeted on conflict control. In addition, the explorations of ERPs in OWs can provide objective indicators of cognitive function improvement, after successful weight loss. For example, a previous study showed that after acute exercise, P3 amplitudes to food stimuli was significantly reduced, compared to those for non-food stimuli, in adolescents with obesity [54].

However, some limitations of the current study should be acknowledged. First, although the sample size was suitable for an ERP study, it was relatively small for a behavioral study, and we only recruited female adults. Future studies should recruit a larger sample (both females and males) to explore the conflict control so that the research conclusions could be better generalized. Second, the present study did not show a causal relationship between overweight and conflict control. Longitudinal studies could be designed to explore the causal relationship between overweight and conflict control. Third, we did not evaluate mental illness in the current study. Future studies could control the impact that mental illness might have on research conclusion. Moreover, the ERP method is not sufficient to understand the spatial dynamics of conflict control. Future studies can use simultaneous EEG–fMRI, which has the advantages of both EEG and fMRI, to explore both temporal and spatial neural responses.

## 5. Conclusions

In conclusion, the present study showed that OWs might be less efficient in monitoring and resolving conflict over food-related and food-unrelated stimuli and might allocate more cognitive resources to food-related stimuli. Moreover, OWs tend to have higher-order processing of food-related stimuli involving motivation or emotion, which is likely to contribute to their difficulty in losing weight. Potential directions for future research will be to explore the causal relationship between overweight and conflict control and to develop intervention strategies targeted on conflict control to address the issue of obesity.

## Figures and Tables

**Table 1:** Demographic information and self-report results.

Variable	OWs (M ± SD)	NWs (M ± SD)
	*n* = 22	*n* = 21
Age	20.55 (2.06)	20.00 (1.48)
BMI **	28.84 (3.86)	20.81 (1.49)
NPS-F *	2.32 (1.04)	1.56 (0.85)
DEBQ-R	35.47 (6.5)	34.07 (10.13)
Hunger	53.64 (28.21)	37.62 (24.01)
Desire	49.77 (26.07)	43.33 (26.52)
Body satisfaction **	22.27 (18.56)	50.95 (23.43)

Note: NPS–F: Negative Physical Self Scale–Fatness concern; DEBQ–R: Dutch Eating Behavior Questionnaire–Restraint Scale; NWs: Normal-weight adults; OWs: Overweight adults (* *p* < 0.05; ** *p* < 0.01).
